# A Cross‐Sectional Study Exploring Patient Experiences, Unmet Needs and Desired Support in Those With Olfactory Dysfunction

**DOI:** 10.1111/coa.70091

**Published:** 2026-01-29

**Authors:** William Ansley, Gabija Klyvyte, Mehmet Ergisi, Natalia Glibbery, Lois Camp, Prajakta Choudari, Mohammed Jawad, Tharsika Myuran, Nikki Garner, Jane Vennik, Lorenzo Stafford, Felix Naughton, Duncan Boak, Carl Philpott

**Affiliations:** ^1^ Rhinology and Olfactology Research Group, Norwich Medical School University of East Anglia Norwich UK; ^2^ Norfolk and Norwich University Hospitals NHS Foundation Trust Norwich UK; ^3^ Norwich Medical School University of East Anglia Norfolk UK; ^4^ Primary Care Research Centre University of Southampton Southampton UK; ^5^ Department of Psychology University of Portsmouth Portsmouth UK; ^6^ School of Health Sciences University of East Anglia Norwich UK; ^7^ SmellTaste Bicester UK; ^8^ Ear, Nose and Throat (ENT) Department James Paget University Hospitals NHS Foundation Trust Great Yarmouth UK

**Keywords:** anosmia, hyposmia, observational, olfactory dysfunction

## Abstract

**Objectives:**

Smell and taste disorders (SATDs) are frequently overlooked despite growing prevalence. They profoundly impact quality of life. Effective therapies for SATDs remain scarce. This survey aimed to assess patient views surrounding the support available at the time of onset of SATDs, and what further support is needed.

**Design and Setting:**

This was a cross‐sectional study consisting of an online survey circulated via the UK charity SmellTaste (known as Fifth Sense until a rebrand in May 2025), exploring features and impacts of SATDs, and opinions surrounding support available, and any further support felt to be necessary by those formally diagnosed or self‐identifying with SATDs.

**Main Outcomes:**

Survey questions were grouped largely into the domains of demographics, features of olfactory issues, impact of disorder, and support network.

**Results:**

A total of 410 participants completed the questionnaire, with the majority being women. When asked how olfactory dysfunction made participants feel, common responses included: ‘sad’, ‘devastated’, ‘angry’, ‘anxious’, ‘depressed’, ‘isolated’ and ‘grief’. Family was reported as the greatest source of support when diagnosed with SATDs (partner/spouse, 36.4%; other family member, 15.1%) followed closely by SmellTaste (34%). Only 2.5% reported General Practitioners as their greatest source of support, with many participants reporting that primary care providers could not provide adequate help, leaving them to seek advice elsewhere (support groups, family, friends and self‐investigation). Further support, covering medical, psychological and social interventions, was considered important.

**Conclusion:**

Given the complexity of issues experienced and the lack of formal support available for people with SATDs, there is a clear need for an intervention addressing unmet support needs.

## Key Points

- Smell and taste disorders (SATDs) are frequently overlooked despite growing prevalence.
- Effective interventions remain scarce.
- Family was reported as the greatest source of support for people diagnosed with SATDs.
- People with SATDs perceived that healthcare practitioners, especially primary care, were unable to offer appropriate and adequate support.
- Research into developing a dedicated intervention addressing unmet support needs is needed for those with SATDs.

## 1 Introduction

### 1.1 Background and Rationale

Smell and taste disorders (SATDs) are frequently overlooked despite increasing prevalence. A meta‐analysis of 25 studies reported an overall prevalence of 22.2% for olfactory dysfunction (OD), with incidence rising with age [1]. Leading causes include sinonasal conditions, post‐infectious olfactory dysfunction (PIOD), post‐traumatic olfactory dysfunction (PTOD), neurological conditions, congenital abnormalities, idiopathic case factors, and toxic or iatrogenic causes [2].

Since the COVID‐19 pandemic, the prevalence of OD in the general population notably increased, driven by a rise in PIOD cases [3]. Meta‐analyses suggest that between 43.0% and 52.7% of COVID‐19 patients experience OD [3, 4, 5], with 10%–15% failing to recover spontaneously [6, 7]. While more recent studies suggest that new strains are less likely to cause OD [8] due to the previous pandemic, the prevalence in the general population remains high [7].

It is estimated between half a million to one million British people experience long COVID. Thirty‐one percent report loss of smell persisting > 12 weeks, and 24% report loss of taste > 12 weeks after COVID [9].

SATDs profoundly affect quality of life, posing significant physical, social, psychological, and emotional challenges [10, 11, 12]. OD poses serious safety risks by impairing the ability to detect hazardous odours (e.g., smoke, gas leaks, spoiled food) [13, 14]. Due to the key sensory input from our chemical senses in the appreciation of flavour, SATDs often diminish enjoyment of food. Individuals with SATDs report higher rates of depression, anxiety, and strained interpersonal relationships [11, 15, 16, 17, 18]. Approximately one‐quarter to one‐third of patients with OD experienced depressive symptoms [19].

A recent qualitative study [10] explored the challenges encountered by those with SATDs including experiences of isolation, relationship difficulties, physical health impacts, and significant costs and challenges associated with seeking help. Dismissive attitudes from healthcare professionals (HCPs) regarding their smell loss and difficulties obtaining appropriate advice and treatment were common. Patients face numerous barriers to accessing healthcare, with HCPs failing to recognise the impact of SATDs or how to manage them [20].

Effective, well‐researched therapies for SATDs remain scarce, limited to olfactory training, with variable evidence and expert opinions surrounding other therapies like steroids and vitamin A drops [21, 22]. Conversely, other chronic conditions have well‐established management protocols, treatment options, and support available (e.g., sensorineural hearing loss).

### 1.2 Aims

This study aimed to explore opinions of those with SATDs in terms of the support available to them at the time of onset of the problem beyond support offered through the smell and taste charity SmellTaste, such as healthcare services and/or support self‐management. The survey was specifically designed to get SmellTaste members' views on a number of areas linked to a National Institute for Health and Care Research (NIHR) Programme Grant application.

## 2 Methods

### 2.1 Study Design

This cross‐sectional study consisted of an online survey. No patient identifiable data were collected but demographics including age and gender were recorded.

### 2.2 Setting

An online survey using Microsoft Forms was distributed via SmellTaste (https://www.smelltaste.org.uk/), the UK charity supporting individuals with SATDs, through their global newsletter and social media platforms (Instagram and Facebook). The survey was piloted by the senior author at the SmellTaste Members.

Conference in November 2023 and then after audience feedback was adapted before data was collected between 24 May 2024 and 26 June 2024.

### 2.3 Participants

Survey participants were individuals with either a formal diagnosis or self‐reported SATD—this was the sole inclusion criterion. There were no exclusion criteria. The survey was open to participants of all ages, worldwide. This survey was not limited to charity members and was open to all individuals.

### 2.4 Variables and Measurements

Data were collected using a questionnaire to assess general experiences of seeking support for their SATD and collect a comprehensive understanding of the respondents' perceptions, experiences, and opinions. They included a combination of question types including single answer, multiple choice, Likert scales, and open‐ended free text responses (Appendix A). Questions were grouped largely into the following domains:

1. Demographics: age, gender
2. Characteristics of SATD: anosmia, hyposmia, ageusia, dysgeusia, parosmia
3. Impact of disorder: emotional, social, functional implications
4. Support network: experiences with existing support networks
5. Opinions on olfactory implants: opinions and perceptions towards olfactory implants (not analysed and reported in this manuscript).

### 2.5 Bias

Different question formats (single answer, multiple choice, Likert scales and open‐ended free text responses) were designed to elicit a variety of responses, mitigating response bias.

### 2.6 Study Size

There was no minimum sample size, as only descriptive (not inferential) analysis was undertaken. Recruitment was based on a convenience sample. The number of active members is around 2000; however, non‐members could also respond to the survey.

### 2.7 Statistical Methods

Data was analysed using descriptive statistics. Microsoft Excel (Microsoft Corporation, headquarters in Redmond, Washington, version 16.0) was used for organising data. IBM SPSS Statistics version 27 (IBM Corporation headquarters in Armonk, New York) was employed for analyses, including analysis of frequencies for multiple choice questions.

Demographic data were summarised using means and standard deviations (standard deviation, SD) or frequencies (%) and percentages as appropriate. Normality was determined using the Shapiro–Wilk test. Data from the Likert scales and other ordinal variables were presented as frequency (%), as well as mean (standard deviation, SD) or median (interquartile range, IQR) depending on the normality of the distribution. Free text responses were analysed by two authors using divisive (top‐down) hierarchical cluster analysis to identify recurring themes and insights related to survey topics. Disagreements between analysing authors were resolved through discussion with a third author.

### 2.8 Ethical Considerations

No ethical approval was required, in line with Health Regulation Authority guidance (https://www.hra‐decisiontools.org.uk/research). All survey data were anonymised.

Participation was voluntary and consent implied by participation. The survey had an introductory page outlining the intended use for data collected, which participants had to consent to before moving to the first question.

### 2.9 Manuscript Preparation

The manuscript was prepared in accordance with Strengthening the Reporting of Observational Studies in Epidemiology (STROBE) guidelines.

## 3 Results

### 3.1 Participants

A total of 410 participants completed the questionnaire.

### 3.2 Descriptive Data

The mean age of respondents was 62.17 years±13.61. 73.8% of participants were women (Table 1).

**TABLE 1:** Demographics and smell and taste status (with the phrasing used in the survey in brackets) of participants.

Demographic details	
Age (mean, SD)	62.17 ± 13.61 (*n* = 402)
Gender, *n* (%)	(*n* = 408)
Women	301 (73.8%)
Men	105 (25.7%)
Prefer not to say	2 (0.5%)
Smell/taste status	(*n* = 406)
Anosmia (no sense of smell)	240 (59.1%)
Hyposmia (reduced sense of smell)	122 (30.0%)
Parosmia (distorted sense of smell)	82 (20.2%)
Ageusia (no sense of taste)	102 (25.1%)
Hypogeusia (reduced sense of taste)	127 (31.3%)
Dysgeusia (distorted sense of taste)	59 (14.3%)

*Note*: Note that for smell and taste status, participants could select one or more answers, or not respond at all. Percentages are calculated as the percentage of total respondents to the smell and taste status question.

### 3.3 Main Results

#### 3.3.1 Characterising Olfactory Dysfunction Amongst Participants

Smell and taste status was reported by 406 participants. Complete anosmia was reported by 59.1% (*n* = 240) and complete ageusia by 25.1% (*n* = 102). A further 30.0% (*n* = 122) reported hyposmia, whilst 31.3% (*n* = 127) reported hypogeusia (Table 1).

#### 3.3.2 Patient Thoughts and Feelings Regarding Olfactory Dysfunction

Divisive hierarchical cluster analysis was used to identify common themes from free text responses. When asked how OD made participants feel, common themes included sadness/depression/grief (*n* = 233, 56.8%), anxiety/vulnerability (*n* = 74, 18.0%), isolation (*n* = 52, 12.7%) and frustration (*n* = 42, 10.2%). Interestingly, 45 participants (11.0%) were equivocal and 7 (1.7%) felt positive about their OD (Table 2).

**TABLE 2:** Frequencies of each theme from cluster analysis of free text responses surrounding patient thoughts and feelings regarding olfactory dysfunction, in total 406 participants responded.

Theme/cluster	*n*	%
Sadness/depression/grief	194	56.8
Anxiety/vulnerability	56	18.0
Anger/annoyance	18	5.1
Isolation	49	12.7
Equivocal	45	11.0
Frustration	34	10.2
Embarrassed	3	0.7
Positive	7	1.7
No response	4	1.0

The impact of OD appeared to depend on cause. For example, many of those who were equivocal had congenital OD, so know no different, and those who felt positive about their OD often suggested it gave them ‘answers’ to what they have experienced. However, those with acquired disorders commonly reported a negative impact on mental health and feelings of embarrassment often related to personal malodour or cooking. Others expressed general feelings of frustration around OD being an ‘invisible disability’ that others may not be able to relate to.

#### 3.3.3 Support for Those With Olfactory Dysfunction

On discovery of their smell/taste disorder, participants reported the greatest support from their partner/spouse (36.4%, *n* = 145), SmellTaste charity (34.4%, *n* = 137), other family members (15.1%, *n* = 60), and ENT specialists (14.8%, *n* = 59). Notably, general practitioners (GPs) and neurologists/other specialists only comprised 2.5% (*n* = 10) and 1.3% (*n* = 5) of responses, respectively.

From free text responses (Table 3), an overwhelming number of respondents felt they had received no support at all (*n* = 183, 44.6%). Commonly, participants indicated that primary care professionals could not offer any support (*n* = 35, 8.5%), so they were left to seek support themselves through support groups/charities (*n* = 86, 21%), family and friends (*n* = 42, 10.2%), and self‐research (*n* = 59, 14.4%). Only a minority of participants reported use of smell training (*n* = 30, 7.3%). A few participants felt relief in having an explanation for their symptoms (*n* = 4, 1.0%).

**TABLE 3:** Frequencies of each theme from cluster analysis of free text responses surrounding what participants found most useful at the time of diagnosis/discovery of their SATD (*n* = 397) and what further support participants would like to see (*n* = 186).

Theme/cluster	*n*	%
*What participants found most useful when they developed their smell and taste disorder*		
No support was available	183	44.6
Other with SATDs	22	5.4
Support groups/charities	86	21.0
Self research	59	14.4
Reported relief from having an explanation for their symptom	4	1.0
Family and friends	42	10.2
Smell training	30	7.3
Medical professionals	35	8.5
No response	13	3.2
*What further support participants would like to see*		
Support finding the cause/getting a diagnosis	17	4.1
More research	17	4.1
Emotional support	4	1.0
More treatment options or information on existing ones	31	7.6
Better access to and/or awareness/knowledge amongst HCPs	66	16.1
Support for safety concerns	11	2.7
Public awareness	16	3.9
More support for the cause of their specific SATD	6	1.5
More support groups or better access to meetings	11	2.7
Disability support	4	1.0
Other	20	4.9
No response	224	54.6

There was a poor response rate for free text regarding further support participants would like (*n* = 224, 54.6% did not respond). Of responders, many felt they were not taken seriously and wanted better access to healthcare professionals (both primary and secondary care), as well as better knowledge amongst practitioners (*n* = 66, 16.1%) and help getting finding a cause/diagnosis (*n* = 17, 4.1%). Others felt there could be more public awareness (*n* = 16, 3.9%) and support for potential safety concerns (e.g., gas, fire, spoiled foods) (*n* = 11, 2.7%).

**FIGURE 1:**
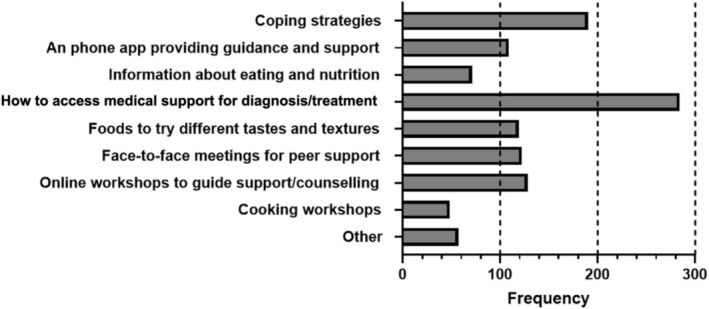
Responses to the question “Which of the following would you have valued most when faced with having no/changed sense of smell/taste?” Note that participants could select one or more answers, or not respond at all. *N* = 395 participants responded.

Participants most valued access to medical support for diagnosis/treatment (71.9%, *n* = 284), coping strategies (48.1%, *n* = 190), online workshops to guide support/counselling (32.4%, *n* = 128), and face‐to‐face meetings for peer support (30.9%, *n* = 122) (Figure 1, *n* = 395).

**FIGURE 2:**
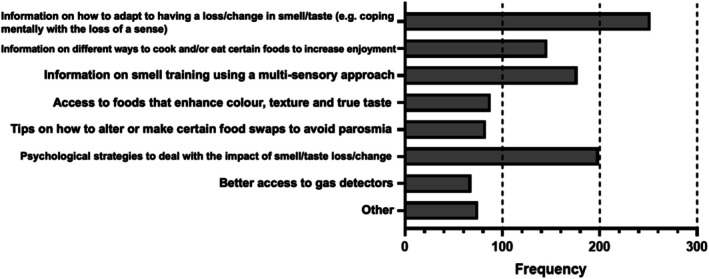
Responses to the question “Which of the following types of support would you like to see or to have seen when the problem arose?” Note that participants could select one or more answers, or not respond at all. *N* = 398 participants responded.

Participants reported interest in general information on how to adapt to having a change to their smell/taste (63.3%, *n* = 252), psychological strategies to deal with the impact (50.0%, *n* = 199), and information on smell training using a multi‐sensory approach (for instance, using different food textures or even different coloured plates to enhance the food flavour/eating experience) (44.5%, *n* = 177). Participants generally indicated a lack of support from healthcare providers in this respect, or felt help was mainly catered towards smell disorders rather than taste (Figure 2, *n* = 398).

In terms of support for food specifically, 36.7% (*n* = 146) reported wanting information on different ways to cook and/or eat certain foods to increase enjoyment, 22.1% (*n* = 88) would have liked access to foods that enhance colour, texture and true taste, whilst 20.9% (*n* = 83) wanted tips on how to alter or make certain food swaps to avoid parosmia. With regards to methods for providing food support, 12.1% (*n* = 46) would like face‐to‐face cooking/food preparation support groups, 9.2% (*n* = 35) a voucher for food packages, and 7.4% (*n* = 28) delivery of bespoke food parcels.

**FIGURE 3:**
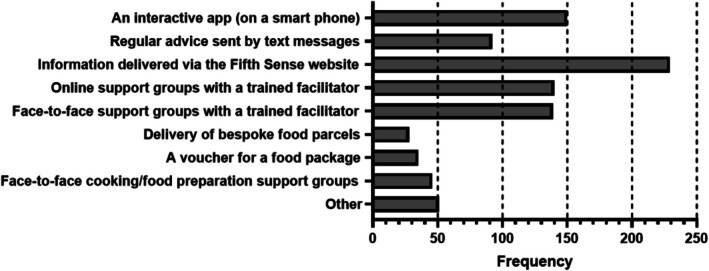
Responses to the question “Which of the following methods would you like to see deliver this type of support?” Note that participants could select one or more answers, or not respond at all. *N* = 379 participants responded.

Participants would like information delivered via the SmellTaste website (60.4%, *n* = 229), an interactive app on a smart phone (39.6%, *n* = 150), online support groups with a trained facilitator (36.9%, *n* = 140), and face‐to‐face support groups with a trained facilitator (36.7%, *n* = 139) (Figure 3, *n* = 379).

## 4 Discussion

### 4.1 Key Results

SATDs have a negative impact on quality of life and common emotions participants felt included sadness/depression/grief (*n* = 233, 56.8%), anxiety/vulnerability (*n* = 74, 18.0%), isolation (*n* = 52, 12.7%), and frustration (*n* = 42, 10.2%). Participants reported being significantly impacted by their SATDs and were particularly affected by altered odour perception and food tasting experiences. Families were the commonest form of support for people with SATDs, and many felt unsupported by primary care.

### 4.2 Interpretation of Results

The demographics of participants and aetiology underlying their SATDs across both surveys was in line with those seen in recent surveys. Likewise, the impact of SATDs on life, especially the associated safety concerns (e.g., being unable to smell gas or rotten food) is very similar to those reported previously [14].

The emotional impact reported by participants mirrored that seen by Erskine and Philpott [10], with anger, sadness, isolation and depression being key themes. Depression amongst SATD patients was highlighted as a key issue by Chen et al. [18] and Croy et al. [19]. Altered odour perception and taste perception were important issues identified by participants of our study, again mirroring Erskine and Philpott's study, which also reported that reduced pleasure of eating led to reduced appetite, weight loss, poorer diet and increased intake of foods with low nutritional value (particularly those high in fat, salt and sugar) [10].

Another similarity between published studies and ours was the perception that HCPs, especially primary care, were unable to provide adequate and appropriate support for those with SATDs [10, 23].

Key new findings from our study highlight key forms of support participants would like to see at the time their disability arose included information on how to adapt to having a change to their smell/taste, psychological strategies to deal with the impact, and information on smell training using a multi‐sensory approach.

Coping strategies (after medical support) were the most requested intervention by participants. The aim of such an intervention would include education and delivery of coping strategies that individuals can tailor to their own needs and choices. This survey serves as preparatory work for identifying the needs of the target population to create such coping strategies.

Participants highlighted the need for better interventions to aid taste, as current help is catered towards smell disorders. Specifically, participants wanted information on different ways to cook and/or eat certain foods to increase enjoyment, access to foods that enhance colour, texture and true taste, and tips on how to alter/make food swaps to avoid parosmia. Studies have previously found that interventions such as colour bowls can influence flavours like saltiness, as well as the desirability of food [24]. Furthermore, improving support frameworks in patients with auditory loss has demonstrated improvements in patient‐reported outcome measures and overall patient experience. Therefore, comparable supportive strategies may yield similar benefits for individuals with SATDs [25].

In terms of support required, participants' responses highlighted the need for a mix of online information and interactive support. Medical, psychological, and social interventions were all deemed important.

At present, SmellTaste offers support in the form of information sheets, support videos and webinars, amongst others. However, as demonstrated by our study, there is very little support available beyond what SmellTaste offers. The scarcity of effective, well‐researched therapies for SATDs, coupled with the poor prognosis, highlights a need for targeted research into developing bespoke and targeted support that addresses the needs highlighted in this paper. Any such research should address the significant impact of SATDs on both physical and emotional health [10], including the prevalence of depression within this population [19].

### 4.3 Strengths and Limitations

This study has several strengths, including responses from participants with a range of olfactory disorders and the fact it covers patient perspectives regarding support for SATDs, something that is previously poorly researched. Whilst providing useful insight, this study has several limitations. Firstly, the study was open to those with self‐reported SATDs; however, previous work has shown that SmellTaste respondents typically reflect the patient demographic seen in specialist smell and taste clinics [2, 14]. Furthermore, not all participants completed all questions, which could have increased potential bias.

The questionnaires were distributed via the SmellTaste newsletter to SmellTaste members, who are likely to already have increased awareness and opinions surrounding SATDs. Selection bias was likely due to the online survey distribution via a UK‐based charity. Bias towards SmellTaste members seeking support and those with internet access in the UK is likely. People whose OD has a significant impact on life are also more likely to have responded. Therefore, the importance of certain support, or willingness to try potential treatments may be overestimated.

Finally, limited information was collected about those completing the survey. It is possible that those engaged with the SmellTaste charity may not be diverse in terms of key demographic characteristics such as socioeconomic status and ethnicity.

## 5 Conclusion

There is a general consensus amongst SATD patients that there is insufficient support available. Future research that considers potential dietary, psychological, and social interventions may help to allow those affected to better manage the physical, emotional, and social sequelae of their SATDs.

## Author Contributions

W.A. and G.K. contributed equally to this paper; therefore, they should be considered as joint first authors. C.P. and D.B. conceptualised the study and were involved in data acquisition. M.E., L.C., and M.J. were involved in data analysis. All authors were involved in the writing of the first draft. W.A. and G.K. wrote and edited the final manuscript, with assistance editing from all other authors. All authors approved the final manuscript.

## Funding

The authors have nothing to report.

## Ethics Statement

No ethical approval was required, in line with Health Regulation Authority guidance (https://www.hra‐decisiontools.org.uk/research). All survey data were anonymised. Participation was voluntary and consent implied by participation. The survey had an introductory page outlining the intended use for data collected, which participants had to consent to before moving to the first question.

## Conflicts of Interest

Professor Carl Philpott is Editor‐in‐Chief/AE of the journal and co‐author of this article. They were excluded from the peer‐review process and all editorial decisions related to the acceptance and publication of this article.

## Supporting information


**Data S1:** Supporting Information.

## Data Availability

The data that support the findings of this study are available on request from the corresponding author. The data are not publicly available due to privacy or ethical restrictions.

## Appendix A Survey Questions Analysed in This Paper

1. Age
2. Sex
  - Man
  - Woman
  - Non‐binary
  - Prefer not to say
3. Do you consider yourself to have …
  - No sense of smell
  - Reduced sense of smell
  - Distorted sense of smell
  - No sense of taste
  - Reduced sense of taste
  - Distorted sense of taste
4. Do you use your sense of smell for your work?
  - Yes
  - No
5. When you discovered you couldn't smell/taste or had a change in smell/taste, how did it make you feel? (e.g., describe an emotion)
  - Free text
6. What things did you find useful in terms of support when you did get your smell/taste disorder? Please suggest anything specific (e.g., internet information, family, healthcare professionals, etc.)
  - Free text
7. Which of the following would you have valued most when faced with having no/changed sense of smell/taste? If you select other, please add comments at the bottom
  - Coping strategies
  - A phone app providing guidance and support
  - Information about eating and nutrition
  - How to access medical support for diagnosis/treatment
  - Foods to try different tastes and textures
  - Face‐to‐face meetings for peer support
  - Online workshops to guide support/counselling
  - Cooking workshops
  - Other (free text)
8. Who did you get the best support from when you discovered your smell/taste disorder? If you select other, please add comments at the bottom.
  - Partner/spouse
  - Other family member
  - Friends
  - GP
  - ENT specialist
  - Neurologist/other specialist
  - SmellTaste
  - Other charity
  - Other person with a SATD
  - Parent
  - Other (free text)
9. Which of the following types of support would you like to see (or to have seen when the problem arose)? If you select other, please add comments at the bottom?
  - Information on how to adapt (get used) to having a loss/change in smell/taste (e.g., coping mentally with the loss of a sense)
  - Information on different ways to cook and/or eat certain foods to increase enjoyment
  - Information on smell training using a multi‐sensory approach (for instance, using different food textures or even different coloured plates to enhance the food flavour/eating experience)?
  - Access to foods that enhance colour, texture and true taste
  - Tips on how to alter or make certain food swaps to avoid parosmia (smell distortion)
  - Psychological strategies to deal with the impact of smell/taste loss/change (e.g., problem solving)
  - Better access to gas detectors
  - Other (free text)
10. Any other support that you would like to see available for people with SATDs? Name something specific you think is not on the list above.
  - Free text
11. Which of the following methods would you like to see deliver this type of support? If you select other, please add comments at the bottom
  - An interactive app (on a smartphone)
  - Regular advice sent by text messages
  - Information delivered via the SmellTaste website
  - Online support groups with a trained facilitator
  - Face‐to‐face support groups with a trained facilitator
  - Delivery of bespoke food parcels
  - A voucher for a food package
  - Face‐to‐face cooking/food preparation support groups
  - Other (free text)

## References

[1] V. M. Desiato , D. A. Levy , Y. J. Byun , S. A. Nguyen , Z. M. Soler , and R. J. Schlosser , “The Prevalence of Olfactory Dysfunction in the General Population: A Systematic Review and Meta‐Analysis,” American Journal of Rhinology & Allergy 35, no. 2 (2021): 195–205. 32746612 10.1177/1945892420946254 PMC13080788

[2] L. Luke , L. Lee , L. Jegatheeswaran , and C. Philpott , “Investigations and Outcomes for Olfactory Disorders,” Current Otorhinolaryngology Reports 10, no. 4 (2022): 377–384. 36465666 10.1007/s40136-022-00438-x PMC9707095

[3] C. S. Von Bartheld , R. Butowt , and M. M. Hagen , “Prevalence of Chemosensory Dysfunction in COVID‐19 Patients: A Systematic Review and Meta‐Analysis Reveals Significant Ethnic Differences,” ACS Chemical Neuroscience 11, no. 19 (2020): 2944–2961. 32870641 10.1021/acschemneuro.0c00460 PMC7571048

[4] J. Y. Tong , A. Wong , D. Zhu , J. H. Fastenberg , and T. Tham , “The Prevalence of Olfactory and Gustatory Dysfunction in COVID‐19 Patients: A Systematic Review and Meta‐Analysis,” Otolaryngology ‐ Head and Neck Surgery (United States) 163, no. 1 (2020): 3–11. 10.1177/0194599820926473 32369429

[5] J. Saniasiaya , M. A. Islam , and B. Abdullah , “Prevalence of Olfactory Dysfunction in Coronavirus Disease 2019 (COVID‐19): A Meta‐Analysis of 27,492 Patients,” Laryngoscope 131, no. 4 (2021): 865–878. 33219539 10.1002/lary.29286 PMC7753439

[6] M. Lechner , J. Liu , N. Counsell , et al., “High Prevalence of Persistent Smell Loss and Qualitative Smell Dysfunction During the Coronavirus Disease 2019 (COVID‐19) Pandemic in the United States: Urgent Need for Clinical Trials,” International Forum of Allergy & Rhinology 13, no. 8 (2023): 1558–1560. 36409559 10.1002/alr.23100 PMC10199954

[7] M. Lechner , J. Liu , N. Counsell , et al., “Course of Symptoms for Loss of Sense of Smell and Taste Over Time in One Thousand Forty‐One Healthcare Workers During the Covid‐19 Pandemic: Our Experience,” Clinical Otolaryngology 46, no. 2 (2020): 451. 33283459 10.1111/coa.13683 PMC8240100

[8] Y. Ota , Y. Yumiya , O. Chimed‐Ochir , et al., “Characteristics of Patients With COVID‐19 and Smell and/or Taste Disorders Depending on Different Virus Strains: A Cross‐Sectional Study in Hiroshima, Japan,” BMJ Open 15, no. 2 (2025): e088377, 10.1136/bmjopen-2024-088377. PMC11848663 39987009

[9] S. A. Gokani , N. H. Ta , A. Espehana , et al., “The Growing Burden of Long COVID in the United Kingdom: Insights From the UK Coronavirus Infection Survey,” International Forum of Allergy & Rhinology 13, no. 8 (2023): 1535–1538. 36479948 10.1002/alr.23103 PMC9877687

[10] S. E. Erskine and C. M. Philpott , “An Unmet Need: Patients With Smell and Taste Disorders,” Clinical Otolaryngology 45, no. 2 (2020): 197–203. 31856420 10.1111/coa.13484

[11] C. M. Philpott and D. Boak , “The Impact of Olfactory Disorders in the United Kingdom,” Chemical Senses 39, no. 8 (2014): 711–718. 25201900 10.1093/chemse/bju043

[12] L. Q. Zou , T. Hummel , M. S. Otte , et al., “Association Between Olfactory Function and Quality of Life in Patients With Olfactory Disorders: A Multicenter Study in Over 760 Participants,” Rhinology 59, no. 2 (2021): 164–172. 33395453 10.4193/Rhin20.403

[13] I. Croy , S. Negoias , L. Novakova , B. N. Landis , and T. Hummel , “Learning About the Functions of the Olfactory System From People Without a Sense of Smell,” PLoS One 7, no. 3 (2012): e33365. 22457756 10.1371/journal.pone.0033365 PMC3310072

[14] L. Lee , L. Luke , D. Boak , and C. Philpott , “Impact of Olfactory Disorders on Personal Safety and Well‐Being: A Cross‐Sectional Observational Study,” European Archives of Oto‐Rhino‐Laryngology 281, no. 7 (2024): 3639–3647. 38396298 10.1007/s00405-024-08529-9 PMC11211102

[15] C. Neuland , T. Bitter , H. Marschner , H. Gudziol , and O. Guntinas‐Lichius , “Health‐Related and Specific Olfaction‐Related Quality of Life in Patients With Chronic Functional Anosmia or Severe Hyposmia,” Laryngoscope 121, no. 4 (2011): 867–872. 21298638 10.1002/lary.21387

[16] T. Miwa , M. Furukawa , T. Tsuhatani , R. M. Costanzo , L. J. DiNardo , and E. R. Reiter , “Impact of Olfactory Impairment on Quality of Life and Disability,” Archives of Otolaryngology – Head & Neck Surgery 127, no. 5 (2001): 497–503. 11346423 10.1001/archotol.127.5.497

[17] R. Jankowski , D. T. Nguyen , M. Poussel , B. Chenuel , P. Gallet , and C. Rumeau , “Sinusology,” European Annals of Otorhinolaryngology, Head and Neck Diseases 133, no. 4 (2016): 263–268. 27378676 10.1016/j.anorl.2016.05.011

[18] B. Chen , C. Benzien , V. Faria , et al., “Symptoms of Depression in Patients With Chemosensory Disorders,” ORL: Journal for Oto‐Rhino‐Laryngology and Its Related Specialties 83, no. 3 (2021): 135–143, 10.1159/000513751. 33756467

[19] I. Croy , S. Nordin , and T. Hummel , “Olfactory Disorders and Quality of Life‐An Updated Review,” Chemical Senses 39, no. 3 (2014): 185–194. 24429163 10.1093/chemse/bjt072

[20] S. Ball , D. Boak , J. Dixon , S. Carrie , and C. M. Philpott , “Barriers to Effective Health Care for Patients Who Have Smell or Taste Disorders,” Clinical Otolaryngology 46, no. 6 (2021): 1213–1222, 10.1111/coa.13818. 34085404 PMC8239785

[21] A. B. Addison , B. Wong , T. Ahmed , et al., “Clinical Olfactory Working Group Consensus Statement on the Treatment of Postinfectious Olfactory Dysfunction,” Journal of Allergy and Clinical Immunology 147, no. 5 (2021): 1704–1719. 33453291 10.1016/j.jaci.2020.12.641

[22] A. B. Addison and C. M. Philpott , “A Systematic Review of Therapeutic Options for Non‐Conductive Olfactory Dysfunction,” Otorhinolaryngologist 11, no. 2 (2018): 61–71.

[23] T. Hummel , K. L. Whitcroft , P. Andrews , et al., “Position Paper on Olfactory Dysfunction,” Rhinology. Supplement 54, no. 26 (2017): 1–30. 29528615 10.4193/Rhino16.248

[24] M. Annette and L. D. Stafford , “How Colour Influences Taste Perception in Adult Picky Eaters,” Food Quality and Preference 105 (2023): 104763.

[25] M. Barker , S. U. Dombrowski , T. Colbourn , et al., “Intervention Strategies to Improve Nutrition and Health Behaviours Before Conception,” Lancet 391, no. 10132 (2018): 1853–1864, 10.1016/S0140-6736(18)30313-1. 29673875 PMC6075694

