# Supplementary material for: A Cross‐Sectional Study Exploring Patient Experiences, Unmet Needs and Desired Support in Those With Olfactory Dysfunction
Source: Clin Otolaryngol. 2026 Jan 29;51(3):466–73. doi: 10.1111/coa.70091 (PMC13050813; doi:10.1111/coa.70091)
Supplement: Supplementary file 1 — Data S1: Supporting Information. [file COA-51-466-s001.docx]

## Appendix 1: Survey questions analysed in this paper

1. Age
2. Sex
   - - Man
     - Woman
     - Non-binary
     - Prefer not to say
3. Do you consider yourself to have …

- No sense of smell
- Reduced sense of smell
- Distorted sense of smell
- No sense of taste
- Reduced sense of taste
- Distorted sense of taste

1. Do you use your sense of smell for your work?

- Yes
- No

1. When you discovered you couldn’t smell/taste or had a change in smell/taste, how did it make you feel? (e.g. describe an emotion)

- Free text

1. What things did you find useful in terms of support when you did get your smell/taste disorder? Please suggest anything specific (e.g. internet information, family, healthcare professionals, etc)

- Free text

1. Which of the following would you have valued most when faced with having no/changed sense of smell/taste? If you select other, please add comments at the bottom.

- Coping strategies
- A phone app providing guidance and support
- Information about eating and nutrition
- How to access medical support for diagnosis/treatment
- Foods to try different tastes and textures
- Face-to-face meetings for peer support
- Online workshops to guide support/counselling
- Cooking workshops
- Other (free text)

1. Who did you get the best support from when you discovered your smell/taste disorder? If you select other, please add comments at the bottom

- Partner/spouse
- Other family member
- Friends
- GP
- ENT specialist
- Neurologist/other specialist
- SmellTaste
- Other charity
- Other person with a SATD
- Parent
- Other (free text)

1. Which of the following types of support would you like to see (or to have seen when the problem arose)? If you select other, please add comments at the bottom?

- Information on how to adapt (get used) to having a loss/change in smell/taste (e.g. coping mentally with the loss of a sense)
- Information on different ways to cook and/or eat certain foods to increase enjoyment
- Information on smell training using a multi-sensory approach (for instance, using different food textures or even different coloured plates to enhance the food flavour/eating experience)?
- Access to foods that enhance colour, texture and true taste
- Tips on how to alter or make certain food swaps to avoid parosmia (smell distortion)
- Psychological strategies to deal with the impact of smell/taste loss/change (e.g. problem solving)
- Better access to gas detectors
- Other (free text)

1. Any other support that you would like to see available for people with SATDs? Name something specific you think is not on the list above.

- Free text

1. Which of the following methods would you like to see deliver this type of support? If you select other, please add comments at the bottom.

- An interactive app (on a smart phone)
- Regular advice sent by text messages
- Information delivered via the SmellTaste website
- Online support groups with a trained facilitator
- Face-to-face support groups with a trained facilitator
- Delivery of bespoke food parcels
- A voucher for a food package
- Face-to-face cooking/food preparation support groups
- Other (free text)
